# 受体酪氨酸激酶AXL在肿瘤靶向治疗耐药中的作用

**DOI:** 10.3779/j.issn.1009-3419.2026.101.01

**Published:** 2026-01-20

**Authors:** Sutong ZHAN, Peilin CHEN, Tangfeng LV, Yong SONG

**Affiliations:** 210000 南京，南京大学医学院附属金陵医院; Nanjing Jinling Hospital, Affiliated Hospital of Medical School, Nanjing University, Nanjing 210000, China

**Keywords:** AXL, 肿瘤, 靶向治疗, 耐药, AXL, Cancer, Targeted therapy, Drug resistance

## Abstract

尽管近年来靶向治疗在肿瘤治疗中取得突破性进展，但耐药性仍是临床面临的重大挑战。已有大量研究证据表明，受体酪氨酸激酶AXL的高表达与肿瘤患者靶向治疗耐药及不良预后高度相关。AXL能够通过改变肿瘤细胞表型、调控DNA损伤修复过程、促进原癌旁路激活和与酪氨酸激酶互作等多种方式，促进耐药发生。临床前及临床研究结果显示，联合抑制AXL能够增强多种靶向治疗疗效，并改善耐药患者预后。本文综述了AXL在肿瘤靶向治疗耐药中的具体作用及靶向AXL治疗策略的研究进展，深入探讨联合靶向AXL在临床应用中的潜在价值，并对其在创新性靶向治疗方案研发和肿瘤精准化治疗中的应用前景进行展望。

## Abstract (en)

Although targeted therapy has made significant advances in cancer treatment throughout these years, drug resistance still remains a major obstacle. Plenty of evidence has proved that abnormal expression of receptor tyrosine kinase AXL is associated with targeted therapy resistance and poor clinical outcomes. AXL drives drug resistance through diverse mechanisms, including altering tumor cell phenotypes, orchestrating DNA damage response process, promoting the activation of bypass signals, or interacting with other receptor tyrosine kinases. Preclinical and clinical studies have demonstrated that combined inhibition of AXL and the other target can enhance the efficacy of various targeted therapies and improve outcomes for patients with drug resistance. This review summarizes recent advances in the specific roles of AXL in targeted therapy resistance and AXL-targeted treatment strategies. It further explores the potential clinical value of combinatorial approaches involving AXL inhibition and discusses future directions for its application in developing novel targeted therapies and advancing precision oncology treatment.

受体酪氨酸激酶（receptor tyrosine kinase, RTK）AXL，也被称为RTK受体UFO，是TAM（TYRO3、AXL和MERTK）RTK家族成员之一。RTK AXL定位于染色体19q13.2，是一个由20个外显子区编码的894个氨基酸构成的多结构域蛋白，包括胞外的两个重复的免疫球蛋白样和III型纤连蛋白样结构域、一个跨膜结构域以及一个胞内的激酶结构域，广泛表达于正常组织和细胞中，包括内皮细胞、小脑、心脏和肝脏^[1]^。而在多种恶性肿瘤中，AXL可出现以表达量增高为主的异常情况，包括急性髓系白血病（acute myelogenous leukemia, AML）、非小细胞肺癌（non-small cell lung cancer, NSCLC）、乳腺癌、胰腺癌、黑色素瘤和肾细胞癌等^[2,3]^。

AXL可通过与配体结合或与其他激酶结合两种方式激活。TAM受体家族成员公认的首要配体为生长停滞特异基因6（growth arrest-specific protein 6, GAS6）。GAS6与AXL结合后与另一个GAS6-AXL复合体组成2:2的同源二聚体，使AXL在细胞内的激酶结构域发生自磷酸化，从而激活下游信号通路。其他已知的能够结合并激活AXL的蛋白包括：配体蛋白S（protein S, PROS1）、TAM RTK家族其他成员、人表皮生长因子受体（human epidermal growth factor receptor, HER/ErbB）家族成员、血小板衍生生长因子受体（platelet-derived growth factor receptor, PDGFR）、间质表皮转化因子（mesenchymal to epithelial transition factor, MET）等^[4,5]^。AXL活化后引起下游磷脂酰肌醇3-激酶（phosphatidylinositol 3-kinase, PI3K）/蛋白激酶B（protein kinase B, AKT）/哺乳动物雷帕霉素靶蛋白（mammalian target of rapamycin, mTOR）、酪氨酸激酶/信号转导及转录激活因子（Janus kinase/signal transducer and activator of transcription, JAK/STAT）信号通路、核因子-κB（nuclear factor-κB, NF-κB）、大鼠肉瘤病毒癌基因（rat sarcoma viral oncogene, RAS）/快速加速纤维肉瘤（rapidly accelerated fibrosarcoma, RAF）/丝裂原活化细胞外信号调节激酶（mitogen-activated extracellular signal-regulated kinase, MEK）/细胞外调节蛋白激酶（extracellular regulated protein kinase, ERK）等下游通路的激活，进而影响肿瘤的增殖、侵袭、迁移以及血管生成等生物学行为^[6,7]^。

## 1 RTK AXL在肿瘤靶向治疗耐药中的作用方式

目前的抗肿瘤治疗研究中，获得性耐药是不良临床预后的重要原因之一，研究表明AXL在抗肿瘤的传统治疗或靶向治疗耐药中起到关键作用。公认的引起靶向治疗耐药的首要原因是获得性继发突变，从而影响酪氨酸激酶抑制剂（tyrosine kinase inhibitors, TKIs）与靶点的正常结合。然而，AXL的基因突变很少见，包括点突变（1.69%）、融合突变（0.04%）、扩增突变（0.21%）或基因缺失（0.09%），其参与获得性耐药的常见方式为过表达^[8]^。AXL主要通过影响表型转化、DNA损伤和修复、原癌旁路激活、与酪氨酸激酶互作等机制引起针对各种靶点的靶向治疗耐药。

### 1.1 表型转化

AXL与肿瘤细胞的上皮间充质转化（epithelial-mesenchymal transition, EMT）进程引发的靶向治疗耐药有关。AXL能够上调波形蛋白的同时下调E-钙黏蛋白，促进头颈部肿瘤细胞发生EMT进而对厄洛替尼耐药^[9]^。此外，Kirsten大鼠肉瘤病毒癌基因（Kirsten rat sarcoma viral oncogene, KRAS）/p53突变的NSCLC发生EMT介导的耐药过程似乎依赖于AXL和MEK，且二者的联合抑制能够预防此类耐药现象的发生^[10]^。在肝细胞癌中，高表达的AXL能够激活波形蛋白和锌指转录因子Slug进而导致索拉非尼耐药^[11]^。

### 1.2 DNA损伤以及DNA损伤应答（DNA damage response, DDR）

AXL过表达可激活ERK/mTOR信号通路，导致G_2_细胞周期检查点蛋白1的招募和激活，从而启动一条平行的DDR通路，促进小细胞肺癌产生WEE1 G_2_细胞周期检查点激酶抑制剂抗性^[12]^。而DDR与AXL抑制剂联合使用能够促进DNA损伤和细胞死亡，增强NSCLC的某些靶向治疗疗效，如共济失调毛细血管扩张Rad3相关蛋白（ataxia telangiectasia and Rad3-related protein, ATR）抑制剂^[13]^。还有研究^[14]^表明AXL能够通过促进RAD18 E3泛素蛋白连接酶的拟素化进而诱导低保真度DNA聚合酶的表达，进行错误倾向的DNA修复，导致二次突变引起的表皮生长因子受体（epithelial growth factor receptor, EGFR）突变阳性肺癌对EGFR-TKI耐药。此外，AXL核转位联动WRN解旋酶相互作用蛋白1（WRN helicase interacting protein 1, WRNIP1）能够维持复制叉稳定性，从而促进HER2阳性乳腺癌的靶向治疗耐药和脑转移^[15]^。

### 1.3 原癌旁路激活

在EGFR突变的NSCLC中，AXL上调是一种EGFR-TKIs获得性耐药的原癌旁路机制，部分原因来自AXL对MAPK/ERK、PI3K/AKT通路的激活^[16]^。近期投入临床使用的KRAS抑制剂在临床KRAS^G12C^突变阳性的NSCLC患者中存在耐药性高发的问题，研究人员^[3]^发现药物通过YAP/GAS6/AXL轴促使AXL过表达并导致下游MAPK/ERK和PI3K/AKT通路激活可能是耐药原因之一，且用药初期联用AXL抑制剂可显著减少耐药现象的发生。

### 1.4 与酪氨酸激酶互作

AXL可通过GAS6依赖或不依赖的方式参与许多其他酪氨酸激酶的交叉互作，进而放大促肿瘤发生、侵袭、耐药信号的作用。其具体的机制包括异源二聚体的形成、交叉磷酸化、提高受体稳定性、替代失活受体等。如在HER2阳性乳腺癌中，高表达的AXL能够与HER2形成异二聚体，激活PI3K/AKT与MAPK/ERK通路，导致曲妥珠单抗耐药，且AXL抑制剂的使用能够逆转该结果^[17]^。

既然AXL能够通过上述多种方式导致肿瘤靶向治疗耐药，这提示我们AXL可能作为一个有潜力的肿瘤治疗靶点，而深入理解这些耐药具体靶分子以及其中关键的信号轴，是揭示耐药机制并设计有效干预策略的重中之重。

## 2 RTK AXL可能导致耐药的具体靶分子

### 2.1 ErbB/HER家族受体

ErbB/HER家族由4个不同的RTK成员组成：ErbB1/EGFR（HER1）、ErbB2（HER2）、ErbB3（HER3）和ErbB4（HER4），它们选择性结合不同的表皮生长因子（epithelial growth factor, EGF）配体，继而发生构象变化，形成同源或异源二聚体并实现磷酸化激活^[18]^。ErbB家族受体的活化能够激活一系列下游信号分子，调控细胞的增殖、分化和迁移，在肿瘤的发生发展中起到关键作用^[18]^。目前，NSCLC、乳腺癌、胰腺癌等多种癌症类型中都可能发现ErbB受体突变导致的扩增或持续激活，成为肿瘤驱动因素，因此靶向ErbB受体是相关突变阳性患者的重要治疗方式。然而，长期靶向治疗后发生的耐药是优良预后的巨大阻力，许多临床前和临床研究正开发新型抗ErbB疗法以克服耐药性，其中，多项研究已证实AXL可能在EGFR和HER2靶向治疗耐药中起到关键作用。

#### 2.1.1 EGFR

早在2012年，研究人员^[19]^就发现AXL的高表达和激活可以导致EGFR突变阳性肺癌出现厄洛替尼耐药。目前研究表明，AXL在乳腺癌、肺癌、头颈部鳞状细胞癌中都可能参与EGFR靶向治疗耐药进程。AXL能够与EGFR形成异二聚体，进而通过EGFR-LATS1/2轴激活YAP导致头颈部鳞状细胞癌和肺腺癌对厄洛替尼耐药，而同时靶向AXL和EGFR能够更有效地抑制肿瘤生长^[20]^。AXL还可通过诱导RAD18发生泛素化进而促进低保真度DNA聚合酶的表达，导致二次突变，从而引起厄洛替尼或奥希替尼耐药，而联合抗AXL治疗可重新杀伤耐药细胞株^[14]^。在EGFR突变的NSCLC中，第三代EGFR-TKI奥希替尼可激活AXL进而重新激活HER3和EGFR及下游通路，导致奥希替尼耐药，而联用AXL抑制剂可在体内及体外使耐药肿瘤对奥希替尼重新敏感或预防耐药发生^[21]^。高表达ZDHHC11的NSCLC可因AXL的高度棕榈酰化，导致其在细胞膜聚集和持续性激活，进而引起下游PI3K/AKT通路活化而致使奥希替尼耐药发生^[22]^。在头颈部肿瘤中，AXL高表达还能够通过促进神经调节蛋白1（neuregulin 1, NRG1）转录引起HER3磷酸化水平上升，进而导致西妥昔单抗耐药发生^[23]^。除了小分子TKIs以外，AXL降解剂YD与吉非替尼或奥希替尼联用可延缓或预防NSCLC中EGFR获得性耐药的发生^[24]^。

#### 2.1.2 HER2

在2009年Liu等^[25]^研究中，AXL激活被发现为HER2阳性乳腺癌对拉帕替尼耐药的潜在新机制。目前，AXL对HER2靶向治疗耐药的促进作用在乳腺癌、胃癌和食管癌中都有报道。具有AXL高表达特征的HER2阳性乳腺癌细胞往往表现出更强的EMT表型和迁移侵袭能力，并且AXL小分子抑制剂R428与拉帕替尼联合使用可在HER2阳性乳腺癌小鼠模型中降低循环肿瘤细胞数量和肺转移负荷^[7]^。还有研究人员^[17]^发现在体内和体外实验中联合使用AXL抑制剂能够逆转曲妥珠单抗耐药。此外，食管鳞状细胞癌中，表现出获得性拉帕替尼耐药特征的肿瘤细胞AXL水平更高，并且拉帕替尼和阿法替尼联合治疗能够在体外显示出协同作用^[26]^。在对阿法替尼耐药的HER2阳性胃癌中，研究人员发现了AXL和MET的表达上升，并且联合使用卡博替尼（针对AXL和MET的多激酶抑制剂）可增加获得性耐药细胞对阿法替尼的体外和体内反应^[27]^。目前发现的AXL导致HER2靶向治疗耐药的分子生物学机制主要为AXL以不依赖配体GAS6结合的方式与HER2形成异二聚体，导致下游PI3K/AKT和MAPK/ERK通路活化，促使耐药发生，而联合使用AXL抑制剂可一定程度上恢复肿瘤对HER2靶向治疗的反应性。

### 2.2 KRAS

KRAS是小GTP酶蛋白质超家族RAS的一员，是关键的细胞“中继开关”，功能是整合生长因子受体的上游信号，并将信号传递到多条效应路径，以驱动细胞生长和增殖^[28]^。突变激活的RAS是人类癌症的重要驱动因素之一，约20%的肿瘤携带RAS突变，其中KARS突变占据了绝大多数（>80%）^[28]^。由于KRAS的蛋白结构特性，靶向KRAS的小分子药物开发困难且疗效不佳，长久以来针对KRAS突变的肿瘤的一个思路是靶向其下游的MEK和其他药物的联合使用。2019年，研究人员^[29]^发现对培美曲塞和抗MEK药物曲美替尼耐药的KRAS突变NSCLC细胞对高度活跃的AXL和内质网应激信号传导有依赖性。此外，Konen等^[10]^研究发现，双重靶向MEK和AXL能够通过抑制KRAS/p53肺癌细胞发生EMT，进而有效预防EMT介导的耐药性发生。近年来，新开发的针对KRAS^G12C^突变的NSCLC的KRAS抑制剂索托拉西布和阿达格拉西布已投入临床使用，但仍存在巨大挑战。已有研究^[3]^表明，AXL信号在KRAS^G12C^突变的NSCLC对索托拉西布和阿达格拉西布的获得性耐药中发挥重要作用，且联合使用AXL抑制剂能够重新增加耐药肿瘤细胞的敏感性。

### 2.3 间变性淋巴瘤激酶（anaplastic lymphoma kinase, ALK）

ALK基因编码胰岛素受体超家族中高度保守的RTK序列，在生理状态下ALK对神经系统的发育和功能至关重要，与配体ALKAL结合后形成二聚体并发生自磷酸化，激活与细胞增殖、存活和分化相关的下游通路^[30]^。而ALK点突变或染色体重排导致的融合突变会导致ALK的异常持续激活，从而驱动NSCLC、胶质瘤、结肠癌、平滑肌瘤等肿瘤发生，而靶向ALK的TKIs抑制剂如克唑替尼、阿来替尼、布加替尼、恩沙替尼、劳拉替尼等成为ALK突变阳性肿瘤患者的重要治疗药物^[30]^。然而，耐药性的出现仍是部分患者预后不佳的重要原因。在2016年，有研究^[31]^报道AXL激活可以通过促进MAPK/ERK通路激活和EMT导致ALK^F1174L^突变的神经母细胞瘤对克唑替尼耐药。此外，AXL对ALK抑制剂耐药的促进作用在NSCLC中也有报道。在克唑替尼耐药的NSCLC患者来源的异种移植小鼠模型中，AXL磷酸化水平上升并且不能被ALK抑制剂逆转，而联合使用AXL抑制剂与克唑替尼单药相比能够更好地抑制肿瘤生长^[32]^。近期，Utsumi等^[33]^发现，部分阿莱替尼耐药的NSCLC患者的肿瘤组织中存在AXL显著高表达，且胸腔积液中发现高水平的GAS6，在体外和小鼠体内实验中证实了AXL过表达的肺癌细胞在补充GAS6的条件下可以促进ALK-TKI耐药发生。

### 2.4 FMS样酪氨酸激酶3（FMS-like tyrosine kinase 3, FLT3）

FLT3是III类RTKs的成员之一，通常表达于造血前体细胞表面，与骨髓间质细胞分泌的FLT3配体结合后激活，在造血系统的维持、造血细胞的增殖和分化中起重要作用^[34]^。FLT3内串联复制（internal tandem duplication, ITD）是AML患者中较为常见的突变。2013年，研究人员^[35]^发现AXL在FLT3-ITD阳性AML患者中高表达并持续激活，同时能够激活FLT3，是FLT3-ITD阳性AML的重要潜在联合靶点。该团队进而证明了对FLT3抑制剂PKC412和AC220耐药的FLT3-ITD阳性AML细胞具有高表达活化AXL的特征，并且AXL抑制剂TP-0903可以逆转这种耐药现象^[36]^。数年后，又有研究^[37]^表明双重靶向FLT3/AXL的TKI吉瑞替尼相比于奎扎替尼能够保持更强的促凋亡作用、更有效地靶向白血病细胞。此外，Seale等^[38]^在研究中观察到FLT3-ITD突变的细胞在FLT3抑制剂（索拉非尼、奎扎替尼、来他替尼、克来拉尼）处理24 h后出现AXL表达量和磷酸化水平上调，进而导致ERK磷酸化信号回升、发生耐药，而联合AXL抑制剂能够在体外和体内阻断此现象，AXL表达量的上升在临床标本中也得到证实。这些结果提示共同靶向AXL是解决FLT3-ITD阳性AML耐药的潜在手段。

随着研究的深入，RTK AXL导致各种具体肿瘤靶点的靶向治疗耐药的具体机制还将进一步拓宽和深入。目前的研究已经表明，联合靶向AXL是提高许多靶向治疗疗效的重要潜在手段，因此发展靶向AXL的有效药物是促进AXL从基础至临床转化的重要环节。

## 3 靶向RTK AXL的研究进展

除了以上讲述的AXL抑制剂与其他靶向治疗联用以更好地抑制肿瘤生长、预防耐药以外，研究者们也在开发新型AXL小分子抑制剂、各种双靶点抑制剂、单抗和抗体偶联药物（antibody-drug conjugate, ADC），以抵抗或预防AXL带来的靶向治疗耐药后果。

### 3.1 小分子抑制剂

目前已上市的选择性AXL小分子抑制剂贝森替尼（Bemcentinib, R428）在克服靶向治疗耐药和改善肿瘤疗效的联合用药中占有重要地位。在2020年Lotsberg等^[39]^的研究中，联合使用贝森替尼能够通过促进肿瘤细胞发生免疫原性细胞死亡而恢复NSCLC对第三代EGFR-TKIs的敏感性。此外，在对靶向程序性死亡受体-1（programmed cell death 1, PD-1）免疫检查点治疗不敏感的STK11/LKB1突变阳性NSCLC中，联用贝森替尼还能够通过促进TCF^+^CD8 T细胞扩增而增强此类患者使用帕博利珠单抗的免疫治疗疗效^[40]^。目前已有针对贝森替尼的临床研究结果发表，不过大多集中于血液系统肿瘤。2023年公布的欧洲骨髓增生异常肿瘤协作组（European Myelodysplastic Syndromes Cooperative Group, EMSCO）II期BERGAMO临床试验^[41]^结果中，研究人员给低甲基化药物治疗失败的骨髓增生异常综合征（myelodysplastic syndromes, MDS）和AML患者服用贝森替尼至少1个周期疗程，其中MDS队列的主要终点应答率为44%[6%完全缓解（complete response, CR），28%骨髓完全缓解（marrow complete response, mCR），6%部分缓解（partial response, PR），6%疾病稳定（stable disease, SD）]，而AML队列的主要终点应答率仅为11%且皆表现为SD。另一新近发表的针对不适用强化化疗的AML患者使用贝森替尼单药或联合低剂量阿糖胞苷的临床研究^[42]^结果显示，使用贝森替尼单药的患者总体客观缓解率（objective response rate, ORR）为14%，其中400/200 mg剂量组的ORR为29%，而联合组的总体ORR达到50%，中位总生存期（median overall survival, mOS）为16.1个月。此外，在针对晚期NSCLC的I期临床研究^[43]^中，患者对贝森替尼的应答率为81%（35% PR, 47% SD），mOS为2.8个月。另一种早期开发的AXL抑制剂度博替尼（TP-0903）能够通过阻止AXL激活下游mTOR信号通路以及DNA损伤后修复，从而克服小细胞肺癌对WEE1抑制剂AZD1775的耐药性^[12]^。

AXL小分子抑制剂的相关研究还在不断地扩展突破。近期，美国食品药品监督管理局（Food and Drug Administration, FDA）批准的用于FLT3突变阳性AML患者的AXL小分子抑制剂吉瑞替尼在临床前研究中表现出对AXL阳性实体瘤，包括食管癌、卵巢癌和胃癌，有着良好抑制效果^[44]^。Han等^[45]^还开发出新型AXL小分子抑制剂布格替尼（Brigatinib），并在临床前研究中证明其能够逆转EGFR突变阳性的NSCLC中发生的AXL介导的奥希替尼耐药。

### 3.2 单抗或ADC

早在2010年，研究人员^[46]^就开发出一种能够阻断GAS6结合的抗AXL单抗W327.6S2，并验证了其在NSCLC和乳腺癌中对肿瘤转移的抑制作用，以及对抗血管内皮生长因子（vascular endothelial growth factor, VEGF）和EGFR靶向治疗的增强疗效作用。近期，Simoni-Nieves等^[47]^构造了同时靶向AXL和EGFR的双重特异性抗体，并初步在体外和动物模型中验证其抗耐药的有效性。

此外，近年来基于AXL单抗开发的针对AXL的ADC也层出不穷。2019年，Genmab公司开发了一款名为Enapotamab vedotin的AXL-ADC，其在临床前研究^[48]^中表现出良好的抗肿瘤活性，主要针对NSCLC，尤其是EGFR突变的奥希替尼耐药NSCLC。在针对免疫治疗耐药的黑色素瘤和肺癌的临床前研究^[49]^中，Enapotamab vedotin联合免疫治疗同样表现出了可观的增强抗肿瘤免疫作用和抗癌潜力。然而，尽管Enapotamab vedotin在I期临床试验中表现出一些疗效，但在公司改变剂量或预测性生物标志物时并没有得到提高，因而没有达到继续开发的标准，公司最终决定停止Enapotamab vedotin的相关研究。ADCT-601是一款偶联了抗AXL抗体和细胞毒性PBD二聚体的ADC。在临床前研究^[50]^中，ADCT-601对一甲基澳瑞他汀耐药的肺癌模型表现出抗肿瘤活性，并且在BRCA-1突变的卵巢癌模型中与聚腺苷二磷酸核糖聚合酶（poly ADP-ribose polymerase, PARP）抑制剂奥拉帕尼有良好的协同作用。ADCT-601目前已在骨与软组织肉瘤中进行了I期临床试验^[51]^，在入组的17例患者中，PR率为11.8%，SD率为47.1%，疾病进展率为41.2%。此外，Pei等^[52]^设计开发的AXL-ADC（AXL02-MMAE）能够靶向肿瘤微环境中M2型巨噬细胞的AXL并显著增强先天免疫和适应性免疫的协调性，能够在耐药NSCLC和三阴性乳腺癌中获得优越疗效。目前，开发AXL-ADC仍需要大量的临床前和临床研究数据来支持其安全性和疗效。

### 3.3 蛋白降解靶向嵌合体（proteolysis-targeting chimeras, PROTAC）

PROTAC是一种双功能性嵌合分子，由一个特异性结合目标蛋白的“弹头”、用于招募E3泛素连接酶的E3配体以及二者之间的连接链组成，是以加速靶蛋白降解为目的的新型癌症治疗策略。目前已有针对AXL的PROTAC处于临床前研究阶段，研究人员^[53]^基于R428的化学结构设计、合成并筛选了两种靶向AXL的PROTAC化合物，并在体外验证其对AXL的高度选择性抑制活性，能够防止肿瘤细胞AXL表达水平的代偿性上升、预防耐药发生。

### 3.4 嵌合抗原受体T（chimeric antigen receptor-T, CAR-T）细胞免疫疗法

当抗AXL抗原受体嵌合至人T细胞表面，即能够将AXL-CAR-T细胞定位至表达AXL的细胞表面并发挥细胞毒性作用。2018年，Wei等^[54]^首次证明了AXL-CAR-T细胞免疫疗法在三阴性乳腺癌异种移植模型中具有抗肿瘤细胞毒性。此外，在高表达AXL的NSCLC中，AXL-CAR-T细胞免疫疗法能够使皮下和肺移植性异种移植物发生消退，且在联用局部放疗（微波消融）后抗肿瘤效果更强^[55]^。还有近期研究^[56]^表明，在大细胞神经内分泌癌（large cell neuroendocrine carcinoma, LCNEC）中，AXL-CAR-T细胞对YAP1高表达亚组的肿瘤细胞具有显著杀伤作用。不过，CAR-T细胞免疫疗法在实体瘤的疗效仍缺乏足够证据，且存在一定的不良反应，目前AXL-CAR-T细胞免疫疗法正在AXL阳性的晚期NSCLC患者中进行I期临床研究（NCT03198052）。

除了靶向AXL单靶点以外，靶向TAM受体家族也是近年来针对AXL的研究热点之一。与AXL同属于TAM受体家族的TYRO3和MERTK成员已被证实与AXL存在密切联系，三者共享配体GAS6及PROS1。MERTK主要在单核、巨噬、自然杀伤（natural killer, NK）细胞及血小板中表达，在肿瘤中与肿瘤微环境的关系更为密切^[57]^。但MERTK的异常高表达也被证实能够促进多种肿瘤形成、增殖、迁移以及抗凋亡，并且MERTK反馈性上调可能是导致靶向AXL耐药的机制之一^[58]^。TYRO3的信号转导通路尚未被充分研究，但有证据表明其与AXL有功能学上的联系，并且TYRO3与AXL可相互促进磷酸化的发生，二者同时高表达能够增强细胞增殖信号^[59]^。在多种肿瘤中，TAM家族成员的高表达常常伴随发生，因此，共同靶向TAM家族酪氨酸激酶成为治疗关键点。基于此，Davra等^[60]^的研究发现，在乳腺癌荷瘤小鼠中同时靶向MERTK及AXL可在抑制肿瘤生长的同时改善肿瘤微环境，有效增强免疫治疗疗效。近期Kong等^[61]^发现了一组大环类MERTK/AXL双靶点抑制剂，对包括NSCLC在内的一些实体瘤有一定的杀伤作用，其团队^[62]^还发现了可同时靶向TYRO3及MERTK的新型小分子抑制剂，并同时对AXL具有一定的选择性抑制效果。

尽管针对AXL的治疗方式已取得以上突破，我们还需要更多的临床前和临床研究来探索这些药物在肿瘤靶向治疗耐药中的实际应用、临床效果、不良反应以及发展瓶颈。

## 4 结语和展望

肿瘤靶向治疗耐药是全世界癌症治疗面临的一项巨大挑战，最近的许多研究表明AXL在这项挑战中占据重要地位，且很可能是一个潜在靶点。除了公认的配体GAS6以外，AXL还与其他多种分子存在直接结合或其他相互作用，如PROS1、EGFR、HER2等，这也是AXL引起相关分子位点靶向治疗耐药的具体作用方式之一。AXL还能够通过影响肿瘤细胞EMT表型、干预DNA损伤修复、激活旁路途径、影响酪氨酸激酶等方式参与到肿瘤靶向治疗耐药进程中。对于AXL引起的获得性耐药，联合使用AXL抑制剂或开发双靶点单抗是可能逆转耐药、提高疗效的解决方案。因此，目前有大量靶向AXL的治疗手段正在开发中，包括已上市的贝森替尼等小分子抑制剂、AXL单抗或ADC药物、靶向TAM家族的药物等，其中一些已进入临床试验阶段。

不过，目前靶向AXL治疗仍存在诸多挑战。虽然已证实AXL高表达常与不良预后有关，但其作为疗效预测标志物并不可靠，例如在AML患者中即使检测出骨髓原始细胞AXL低表达，患者仍可能在AXL抑制剂的联合化疗中获益^[63]^，这也提示靶向AXL的治疗手段需要更多维的预测模型，而目前的临床应用缺乏可靠的预测性生物标志物。并且，由于AXL还与另外两个家族成员共享配体，在信号传导和生理功能上存在重叠，前文也介绍了靶向其中单一激酶可能导致其他成员的上调代偿，导致对单一激酶抑制剂的耐药，这提示了开发泛TAM家族抑制剂的必要性，但因其广泛的正常生理功能又大大增加了毒性风险。

此外，联合用药仍有许多因素需要纳入考虑范围。在肿瘤微环境中，AXL能够诱导免疫抑制性M2型巨噬细胞极化，抑制T细胞功能，并促进调节性T细胞的活性，从而共同营造一个“冷肿瘤”免疫微环境，因此高表达AXL往往预示着免疫治疗疗效不佳^[64]^。已有研究^[40]^表明靶向AXL能够显著增加肿瘤内细胞毒性T细胞的浸润，逆转免疫抑制状态，提高免疫治疗疗效。然而，除了肿瘤细胞本身及其周围微环境以外，AXL激酶信号活性在许多正常生理生化过程中占据重要地位，包括固有免疫、血小板聚集、血管稳态维持等方面。因此，在临床应用时，必须谨慎评估其潜在的副作用，包括但不限于免疫相关不良反应、代谢异常及出血风险等^[65,66]^。

未来的研究需进一步拓展细化AXL可能导致靶向治疗耐药的具体分子类型和机制探究，并且在AXL靶向药物的联合使用方面，还需要大量的临床研究数据来探索其不同耐药靶分子中疗效最佳的具体方案。对于AXL可能导致的脱靶效应和药物毒性，聚焦于输送AXL抑制剂抵达肿瘤位点以减少系统毒性的药物包装技术，如新型纳米给药系统，或依赖于肿瘤独特酸性微环境等条件激活的ADC抗体，是未来研究的潜在可行方向。

总而言之，靶向AXL是一个有前景的克服靶向治疗耐药的新策略，随着我们对依赖于AXL耐药机制的不断发掘以及靶向AXL药物的临床前开发和临床试验的进展，未来将为肿瘤患者提供潜在新方案。
